# Biological evaluation of the modified nano-amorphous phosphate calcium doped with citrate/poly-amino acid composite as a potential candidate for bone repair and reconstruction

**DOI:** 10.1007/s10856-020-06482-7

**Published:** 2021-01-25

**Authors:** Xiaomei Wang, Dechuan Zhao, Haohao Ren, Yonggang Yan, Shuyang Li

**Affiliations:** 1 College of Physics, Sichuan University, Chengdu, 610064 China; 2 Collaborative Innovation Center of Tissue Repair Material of Sichuan Province, College of Life Sciences, China West Normal University, Nanchong, 637009 China

## Abstract

Large numbers of research works related to fabricating organic–inorganic composite materials have been carried out to mimic the natural structure of bone. In this study, a new modified n-ACP doped with citrate (n-ACP-cit)/poly (amino acids) (PAA) composite (n-ACP-cit/PAA) was synthesized by employing high bioactive n-ACP-cit and the biodegradable and biocompatible PAA copolymer. Its basic structure was characterized by X-ray diffraction spectroscopy, Fourier transformed infrared spectroscopy, and X-ray photoelectron spectroscopy. Moreover, the degradability, bioactivity, biocompatibility, and osteoconductivity of n-ACP-cit/PAA composite were evaluated in vitro and in vivo, using simulated body fluid (SBF) solution soaking test, mouse bone marrow mesenchymal stem cells proliferation and differentiation, morphological observation test, expression of genes associated with osteogenesis, and bone defect model repair test, respectively. The modified n-ACP-cit/PAA composite exhibited a much higher weight loss rate (36.01 wt.%) than that of PAA (23.99 wt.%) after immersing in SBF solution for 16 weeks and the pH values of local environment restored to neutral condition. Moreover, cells co-culturing with composites exhibited higher alkaline phosphatase activity, more calcium nodule-formation, and higher expression levels of osteogenic differentiation-related genes (Bmp-2, Colla I, OCN, OPN, and Runx-2) than that of PAA. Furthermore, the bone defect model repair test revealed that the composite could be intimately incorporated with the surrounding bone without causing any deleterious reaction and capable of guiding new bone formation. Together, these results indicated that the new modified bone repair n-ACP-cit/PAA composite material with specific characteristics may be designed for meeting diverse requirements from biomedical applications.

## Introduction

Natural bone is an inorganic–organic composite material with high fine and excellent bioactivities assembled from inorganic and organic matter [1]. Among the inorganic matter, tricalcium phosphate, calcium sulfate, hydroxyapatite, and other inorganic materials occupy a large proportion in bone tissue and bone matrix. But to some extent, they are limited due to high crystallinity, slow degradation, or acidic degradation environment [2]. In recent years, more researchers found that nano-amorphous calcium phosphate (n-ACP) can be used as a carrier to transfer calcium and phosphorus ions that are necessary for mineralization in the early stage of bone formation during its transformation process [3]. Moreover, it is found that the n-ACP owns the higher reactivity, biological activity, and biocompatibility due to its larger specific surface area, but n-ACP is unstable. As our previous work [4], the results suggested that we successfully obtained the stable n-ACP doped with citrate (n-ACP-cit). Hence, the stable n-ACP-cit could be considered as a promising filling material owing to their biocompatible and bioactive properties in bone repair and regeneration.

For organic matter, synthetic poly (amino acid) (PAA), fabricated by an in situ melting method has already widely used in surgical sutures [5], artificial skin [6], and drugs or genes delivery system [7] due to the structure similar to human polypeptide collagen, and the copolymer is metabolized by human tissues and discharged in vitro with carbon dioxide and water, which has good biocompatibility [8]. Though it has excellent properties, from the view of bionics, the single polymer has seldom been used directly as bone repair materials [9]. Previous works have proved that the presence of bioactive particles could endow the polymer with bioactive behaviors [10, 11]. Thus, the most modified bone repair materials were fabricated by combining the bioactive inorganic Ca-contained compounds [12–14] and the polymer matrix together.

So, the biomimetic modified n-ACP-cit/PAA composite was designed in this study. The n-ACP-cit as an inorganic additive endowed the composites with excellent bioactivity and osteogenic activity, and the PAA copolymer as the matrix provided the controllable degradation properties for the composites and solved the problem of n-ACP-cit powder in molding. The modified n-ACP-cit/PAA composites were prepared through melt extrusion, a convenient and effective way to prepare a composite. This study was mainly focused on the preparation and characterization, evaluation of degradation, bioactivity, cytocompatibility, and osteogenic activity in vitro and in vivo of the modified n-ACP-cit/PAA composites. More broadly, we expected that this new composite material would retain the desirable properties of the PAA copolymer and the n-ACP-cit powder, also would be an ideal bone repair material.

## Materials and methods

### Preparation of n-ACP-cit/PAA composites

The n-ACP-cit/PAA composites were synthesized using melt extrusion method. Firstly, n-ACP-cit powder was prepared according to our previous publication [4]. Secondly, PAA copolymer was obtained as followings: 6.56 g l-hydroxyproline, 8.91 g l-alanine, 36.09 g γ-aminobutyric acid, and 65.59 g 6-aminocaproic acid (Shanghai Experimental Reagents Co., Ltd., China) were added to a 250.00 ml reaction flask. Subsequently, 50.00 ml deionized water used as a dispersant and 0.50 ml orthophosphoric acid (Chengdu Kelong Chemical Industry Company, China) aqueous solution (V_H3PO 4_: V_H2O_ = 1:1) used as a catalyst were also added into the above reactant. The reaction flask was heated and maintained at 190 °C until the water was evaporated completely, and then the system was kept at 210, 220, and 230 °C for 1, 2, and 4.5 h, respectively. Among the whole reaction process, a continuous flow of nitrogen gas was applied to avoid undesirable oxidation reaction. In addition, after cooling down to room temperature, the obtained PAA copolymer was shattered into granules further use. According to the mass ratios (n-ACP-cit: PAA were 0:100, 15:85, 30:70, 45:55, and 55:45), the modified composites were fabricated by the method of melt extrusion at 140–160 °C and the frequency is 50 Hz. Here the fabricated modified composite materials were named as PAA, 15%n-ACP-cit/PAA, 30%n-ACP-cit/PAA, 45%n-ACP-cit/PAA, and 55%n-ACP-cit/PAA without repeating in later, respectively. Finally, one part of the materials was shattered into granules with the dimension of 2500–3200 μm for degradation and animal experiment and the other part was cut into slices with the dimension 0.5 × 0.5 × 0.1 mm for evaluation of biological performance assays. The pure PAA was used as the control group. For the experiment in vivo, firstly, the n-ACP-cit/PAA composite (2500–3200 μm) were soaked in anhydrous ethanol for 10 min, then ultrasonic cleaning was conducted for 5 min, thirdly, deionized water was used for alternating washing for three times, and freeze-dried to the constant weight. Finally, the implanted composite materials were screened (particle size 1.18–2.00 mm), divided, numbered, and sterilized with ethylene oxide. After sterilization, the samples were placed in a ventilated place for a week, and the residual gas was volatilized.

### Characterization

The composition and structure of the dried n-ACP-cit/PAA composites were characterized by the following methods.

X-ray diffraction spectroscopy (XRD) patterns (Cu, kα1 line, 40 kV, 26 mA) of the powder samples were obtained at a scanning rate of 2°/min 10–60° (EMPYREAN, manufactured Panalytical B.V. of the Netherlands). The spectra were obtained in a Fourier transformed infrared spectroscopy (FT-IR) (Nicolet 6700, Thermo Fisher Scientific, Waltham, MA, USA), with resolution of 4 cm^−1^, at 400–3600 cm^−1^. The surface chemical composition of each samples was analyzed by X-ray photoelectron spectroscopy (XPS) (AXIS Ultra DLD, English Kratos Company). A Japanese scanning electron microscope (SEM) (JEOL JSM5600LV) was used to observe the morphologies of the deposited apatite before and after on the surfaces of the samples. The test voltage was 20 kV and a 10-nm layer of gold was sputtered on the surfaces. Also, the element composition was analyzed via an energy-dispersive X-ray detector (EDX, HORIBA Company, Kyoto, Japan) mounted on an SEM.

### Mechanical property

The mechanical properties (including the compressive strength, tensile strength, and bending strength) of the composites were tested by a mechanical testing machine (Instron, Norwood, MA, USA) equipped with a 10 KN load cell. The mechanical testing specimens were molded into the size of 5 × 5× 5 mm^3^ for compressive strength, 80 × 10 × 4 mm^3^ for bending strength, 80 × 4 × 2 mm^3^ dumbbell-shaped strips for tensile strength. The samples were tested with a controlled speed of 5 mm/min at room temperature, and the load was applied until the specimens were compressed either approximately 25% of their original height or until the sample collapsed. Three replicates were tested and the results were expressed as mean ± standard deviation (SD).

### Degradation in vitro

Degradation tests in vitro were carried out in simulated body fluid (SBF) (pH 7.40) solution, which was prepared according to a previously reported protocol [15]. The degradation rate of the samples was determined by measuring its mass loss ratio. That is, the initial mass (M_0_) was weighed accurately after the samples were dried in a lyophilizer until constant mass. According to the national standard of YY/T0474-2004, the specimens were soaked in SBF solution with a solid/liquid mass ratio of 1 g/30 ml in polyethylene tubes that were incubated at 37 °C in a water bath shaking at 70 rpm for up to 16 weeks with SBF weekly refreshment. At certain time periods (i.e., 1, 2, 3, 4, 6, 8, 12, and 16 weeks), the specimens were removed from the liquid, and the samples was washed by deionized water followed by drying in the lyophilizer until obtained the constant mass (M_i_). Finally, the mass loss ratio of the samples was calculated using the following equation:

Mass loss rate (wt.%) = (M_0_ – M_i_) / M_0_ × 100%

At the same time, the degradation solutions were measured by pH meter at specific time periods (1, 2, 3, 4, 6, 8, 12, and 16 weeks) and each sample was in triplicate, and then took their mean values.

### Apatite mineralization

Surface biomimetic mineralization of composites (0.5 × 0.5 × 0.1 mm) was assessed in vitro using SBF solution (pH = 7.40) [16] in the shaking water bath with constant temperature of 37 °C, 100% humidity, and 70 rpm for 7 days. The ratio of the surface area of the specimen to the volume of the SBF solution was 0.1 cm^2^/ml [17]. The SBF solution was refreshed every other day to maintain the ionic concentration and pH values during the biomimetic mineralization process. After incubation for 7 days, the specimens were washed with deionized water to remove any soluble inorganic ions and air-dried. The composition and morphology of the deposited layer on the surface of each specimen were analyzed using SEM equipped with EDX, respectively.

### Biocompatibility in vitro

#### Cell culture

The mouse bone marrow mesenchymal stem cells (mBMSCs, type RL-12424) supported by Chinese Academy of Sciences, Shanghai, China, which possesses excellent differentiation capabilities, are potential candidates for assessing the biocompatibility in vitro of the composites. The cells were incubated in 25 cm^2^ culture flask in alpha Modified Eagle’s Medium (a-MEM, HyClone, Thermo Fisher Scientific, Waltham, MA, USA) supplemented with 10% fetal bovine serum (Gibco, Thermo Fisher Scientific, Australia) and 1-vol.% penicillin/streptomycin solution (Gibco, Life Technologies, USA) until the cells overgrew nearly 80% of the culture flask. The culture medium was replenished every other day and incubated at 37 °C under an atmosphere of 100% humidity with 5% CO_2_. All of the samples were sterilized using ethylene oxide.

Prior to cell seeding, samples were immersed in α-MEM for 24 h with a concentration of 200 mg per ml of α-MEM firstly. Then the mixtures were centrifuged at 1200 rpm for 4 min and the supernatant were collected through by a sterile permeable membrane with a pore size of 0.22 μm. After that the leaching liquor was diluted into different concentrations (50, 25, 12.5, and 6.25 mg/ml). The cells were cultured with the leaching liquor that mentioned above.

#### Cell proliferation and morphology

Cell proliferation was conducted with mBMSCs by culturing the leaching liquor of PAA, 15%n-ACP-cit/PAA, 30%n-ACP-cit/PAA, 45%n-ACP-cit/PAA, and 55%n-ACP-cit/PAA composites together. Namely, 100-μl/well cell suspensions were added to 96-well plates and incubated under a 100% humidified atmosphere with 5% CO_2_ at 37 °C. Then, the leaching liquor was used for cell proliferation assays, and refreshed every other day. At predetermined time (1, 4, and 7 days), 10 μl Cell Counting Kit 8 solution (CCK-8, Keygen Biotech, Nanjing, China) was added to the each well, and then incubated in the cell incubator for another two hours. The optical density (OD) of each well at 450 nm was evaluated using a microplate reader (Multiskan FC, Thermo Fisher Scientific, Shanghai, China). At last, the cell relative growth rate (RGR) was calculated as the percentage ratio of OD_leaching_ / OD_blank_ × 100%. Five samples per group were tested for the statistical analysis and the group without adding the leaching liquor was used as the blank control.

In order to observe the cell morphology, the cells at a density of 3 × 10^4^ cells/ml in 24-well plate were fixed with 4% paraformaldehyde at room temperature for 10 min after 3 days of cultivation, and then the cells were permeabilized with 0.5 vol. % Triton X-100 (Invitrogen, Carlsbad, CA, USA) solution. The extra paraformaldehyde solution and Triton X-100 solution were washed away with preheated phosphate buffered saline (PBS, Gibco, Thermo Fisher Scientific, Waltham, MA, USA) solution twice again. Prior to the fluorescence inverted microscope (Nikon Eclipse Ti-U, Nikon Instruments (Shanghai) Co., Ltd., Shanghai, China) analysis, the fixed cells were stained with phalloidin (FITC, Xiangsheng Biotechnology Co. Ltd., Shanghai) for 30 min and 4’, 6-diamidino-2-phenylindole (DAPI, Solarbio Science & Technology Co., Ltd., Beijing) for 40 s, sequentially.

#### Osteogenesis of mBMSCs

The basic inducing medium was supplemented with 0.1 μmol/l dexamethasone (Sigma-Aldrich, USA), 50 mg/l l-ascorbic acid (Sigma-Aldrich, USA), and 10 mol/l β-sodium glycerophosphate (Sigma-Aldrich, USA). The n-ACP-cit/PAA composites were co-cultured with mBMSCs and the cells were seeded at a density of 5 × 10^4^ cells/ml in 24-well plate. After 24 h of adherence, the culture medium was replaced by the inducing mediums.

At the set time point, the cells were harvested at 4, 7, and 14 days and rinsed with PBS (Gibco, Thermo Fisher Scientific, Waltham, MA, USA) for three times, and the cell at each well was lysed by the 1 vol.% Triton X-100 buffer solution. Moreover, the cells were frozen at −80 °C for 10 min and thawed to room temperature for three time to lyse completely. The alkaline phosphatase (ALP) activities of the mBMSCs were measured using the mouse ALP Elisa Kit (MIbio, Shanghai, China) and a Bicinchoninic Acid Protein Assay Kit (Solarbio Beijing Co., China), following the manufacturer’s guidelines at 4, 7, and 14 days.

The calcium nodule of the cells was stained with 1 wt.% Alizarin Red S (Cyagen Biosciences Co., Ltd., Guangzhou, China) after 14 and 21 days of inducing cultivation to observe the osteogenesis activity of mBMSCs. The mineralized calcium nodules in the 24-well plates were observed through an optical microscope (Nikon Eclipse Ti-U, Nikon Instruments (Shanghai) Co., Ltd, Shanghai, China). Moreover, as to cetylpyridinium chloride can dissolve the stained red calcium nodules and changed into a purple transparent solution after dissolution. The OD values were tested at its maximum absorption wavelength (620 nm) to quantitatively reflect the deposition of calcium nodules [18].

The genes related to osteogenic differentiation, such as Bone morphogenetic protein 2 (Bmp-2), Collagen type I (Colla I), Osteocalcin (OCN), Osteopontin (OPN), and Runt-related transcription factor 2 (Runx-2), were analyzed via a real-time fluorescent quantitative polymerase chain reaction (RT-qPCR, Stepone plus, ABI). The mBMSCs were seeded and cultured in osteogenic medium as described previously. The samples were harvested on 4, 7, and 14 days, respectively. The total RNA of the mBMSCs grown on the samples was isolated using the TRIZOL reagent (Ambion Company, USA). The sequences of the forward and reverse primes are shown Supplementary Table 1. Reverse transcription was performed following the protocol of the Revert Aid First Strand cDNA Synthesis Kit (Fermentas, Thermo Scientific Molecular Biology, Pittsburgh, PA, USA).

### Biocompatibility in vivo

In accordance with the strict guiding opinions on treating experimental animals issued by the Ministry of Science and Technology of the People’s Republic of China in 2006 [19], ten New Zealand white rabbits (Sichuan Aolijin company, Chengdu, China) with a mean body weight of 2.8 kg (range:2.5–3.0 kg) were prepared for undergoing surgery by carrying the unchanged feeding conditions before and after operation. The experimental group was randomly divided into the implant group (PAA, 15%n-ACP-cit/PAA and 45%n-ACP-cit/PAA) and the blank group (without any material implantation), the photos of implanted materials and the operation process in femoral condyle are listed in Supplementary Figs. 1 and 2, respectively.

**Fig. 1:**
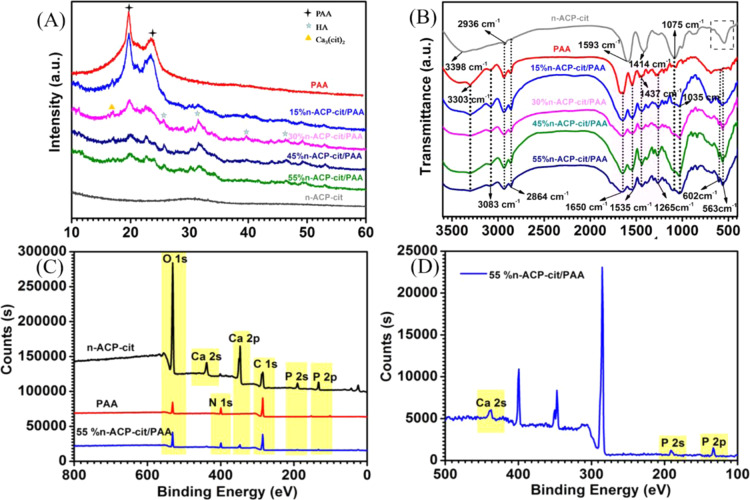

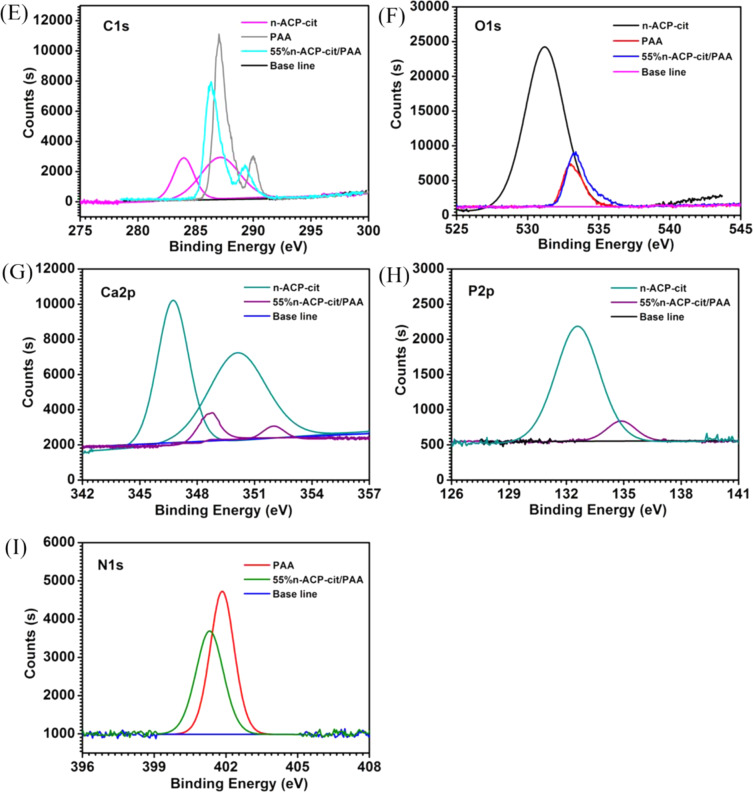
**A** The XRD patterns of PAA and n-ACP-cit/PAA composites. **B** The FT-IR spectra of PAA and n-ACP-cit/PAA composites. **C** The XPS spectra of PAA and n-ACP-cit/PAA composites. **D** The enlarged Ca2s, P2s, and P2p regions of 55%n-ACP-cit/PAA; the patterns of C1s (**E**), O1s (**F**), Ca2p (**G**), P2p (**H**), and N1s (**I**) after fitting

**Fig. 2:**
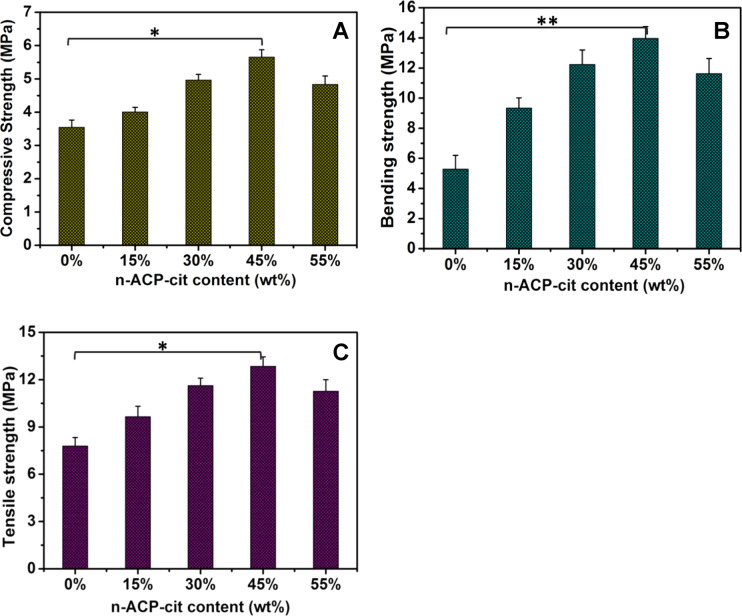
Mechanical properties of the composites with different content of n-ACP-cit (**A** compressive strength, **B** bending strength, **C** tensile strength, *p < 0.05, * *p < 0.01, compared with PAA group)

For the implantation in bone, the New Zealand rabbits were anesthetized by intravenous injection of pentobarbital sodium (30 mg/kg, the mass of the liquid to rabbit weight ratio) in the ear margins of their ears [20]. In brief, their limbs were fixed and a vertical incision was made in the middle part of the lateral femoral condyle to separate the skin and muscles in order to expose the femoral condyle. Then, a hole with a bone defect of 6 × 8 mm was drilled with an electric drill parallel to the joint surface. The femoral condyle defect sites were filled and compacted with PAA, 15%n-ACP-cit/PAA and 45%n-ACP-cit/PAA composite materials. Moreover, the wound was closed with resorbable sutures, and the penicillin was injected in intramuscular for 3 consecutive days to prevent infection. The weight, activity, and feeding conditions of the animals after the operation were observed on time to check whether they were infected or not. After operation, calcein, xylenol orange, and calcein were injected into the ear vein of rabbits at the set time points respectively to investigate the osteogenesis at each time point. The corresponding injection dose and other parameters are shown in Supplementary Table 2. At the predetermined points, implants were fixed with 4% formaldehyde, embedded by polymethyl methacrylate after gradient ethanol dehydration. Meanwhile, 1% methylene blue (Sigma) and 0.3% basic fuchsin (Sigma) solution was stained on the samples to observe whether there was foreign body rejection, immune reaction, and related osteogenic conditions in the bone tissues around the material. Finally, histological observation was performed with light microscopy and the histological slides were scanned with a scanner (Dimage Scan Elite 5400 II, Konica Minolta).

### Statistical analysis

The results of the relative cellular viability, ALP activity, and RT-qPCR were performed with a one-tailed Student’s *t*-test. The EDX results are presented as the mean ± SD. The statistical significance was set at a value *p* < 0.05.

## Results

### Characterizations of PAA and n-ACP-cit/PAA composites

The XRD patterns of PAA and n-ACP-cit/PAA composites are shown in Fig. 1A. The peaks presented at 20.0° and 23.8° were attributed to PAA. With the increasing content of n-ACP-cit, the diffraction peaks of composites were not dramatically affected due to amorphous structure of n-ACP-cit. Nevertheless, the intensity of the two diffraction peaks of PAA in the composites substantially decreases, which was due to the interaction between n-ACP-cit powder and the PAA chains resulting in reducing the regularity of PAA molecular chain.

The characteristic absorption peaks (Fig. 1B) related to the stretching vibration of PAA were observed at 3438 cm^−1^ (-N-H-), 2927 cm^−1^ (-CH_2_-), and 2858 cm^−1^ (-CH-). The peaks at 1639 and 1549 cm^−1^ correspond to amides I and II, respectively. In spectrum of n-ACP-cit, all the typical peaks of n-ACP-cit were observed. The characteristic bands at 1593 and 1409 cm^−1^ were the anti-symmetrical and symmetrical vibrations of the carboxyl group of citrate, and peaks at 1078 and 546 cm^−1^ were attributed to the vibration bands of (PO_4_)^3–^, indicating the existence of citrate and phosphate within the as-prepared composite material.

As we can be seen from Fig. 1C–I, n-ACP-cit /PAA composite materials mainly contain C, O, Ca, P, and N elements, 285.0, 400.0, and 532.0 eV are respectively the electron binding energy of carbon (C1s), nitrogen (N1s), and oxygen (O1s) [21], and 345.6, 439.87, 132.96, and 191.86 eV are respectively the electron binding energy of Ca2p, Ca2s, P2p, and P2s [22]. In detail, from the energy spectrum of each element, we can know that in N1s spectrum the electron binding energy of C-N moved from 401.84 eV in PAA to 401.30 eV [23], 531.22 eV and 533.01 eV were the electron binding energies of C=O/O-C=O and C-O-C/C-O-H in O1s spectrum, respectively. Moreover, the electron binding energies of Ca2p3/2, Ca2p1/2, and P2p were 348.69, 351.98, and 34.90 eV.

### Mechanical property

Figure 2 showed the effect of the mass percentage of n-ACP-cit on the mechanical property of the composite. It could be seen that with the increasing of n-ACP-cit in the composite, the tensile strength, bending strength, and compressive strength were increasing gradually. However, when the mass percentage was 55 wt.%, the mechanical properties began to deteriorate, and the highest compressive strength, bending strength, and tensile strength were 5.65 ± 0.22, 13.97 ± 0.79, and 12.85 ± 0.59 MPa of the composites when the content of n-ACP-cit was 45 wt.%. The above results showed that the composites had good biomechanical property, among which, the 45 wt.% n-ACP/PAA had the best mechanical property.

### Weight loss and pH value

**Fig. 3:**
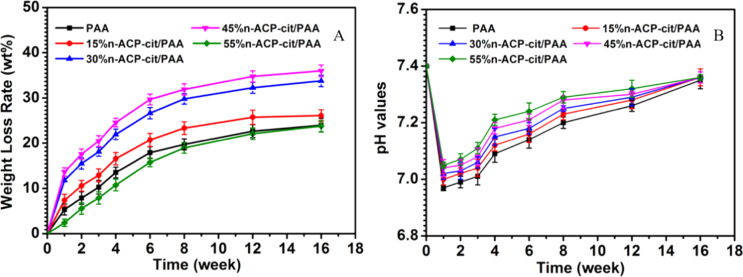
The weight loss rate (**A**) and pH value changes (**B**) of PAA and n-ACP-cit /PAA composites soaked in SBF solution for 16 weeks

The weight losses of the PAA and n-ACP-cit /PAA composites immersed in SBF solution for certain intervals are shown in Fig. 3A. With the extension of degradation cycle, the weight loss rate of composites increased gradually. The weight loss rates of PAA, 15%n-ACP-cit /PAA, 30%n-ACP-cit/PAA, 45%n-ACP-cit/PAA, and 55%n-ACP-cit/PAA composites were 23.99, 26.11, 33.77, 36.01, and 23.79 wt.% after soaking for 16 weeks, respectively, and remained basically unchanged at the sixteenth week.

The pH values of PAA and the composite degradation solution showed a trend of decreasing firstly and then increasing (Fig. 3B). As the content of n-ACP-cit in the composite increased gradually, the pH values of degradation solution also increased gradually. On the whole, with the gradual extension of degradation cycle, the pH values of composite materials were stable at about 7.40, which is the optimal pH range required by human physiological environment.

### Apatite mineralization

**Fig. 4:**
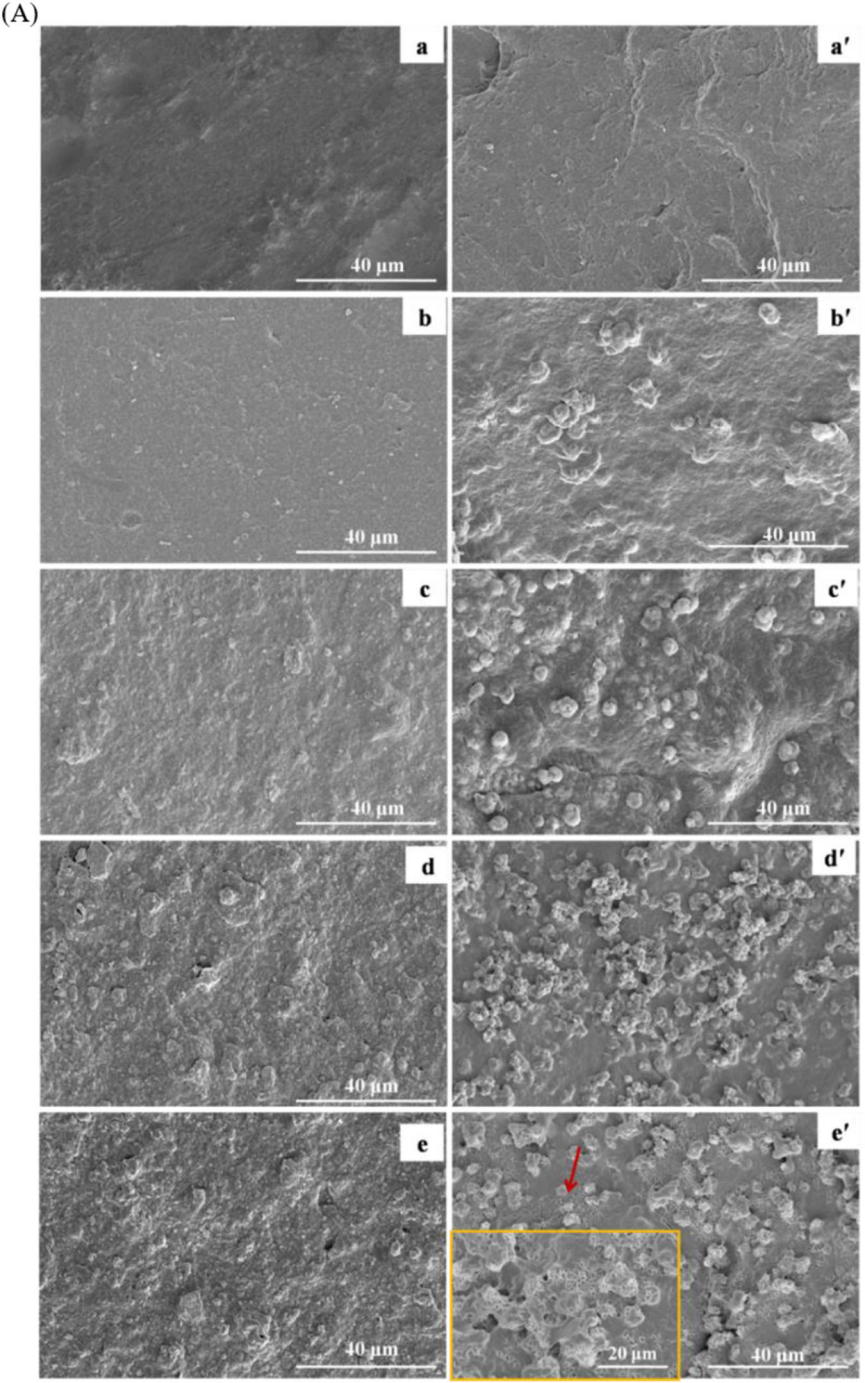

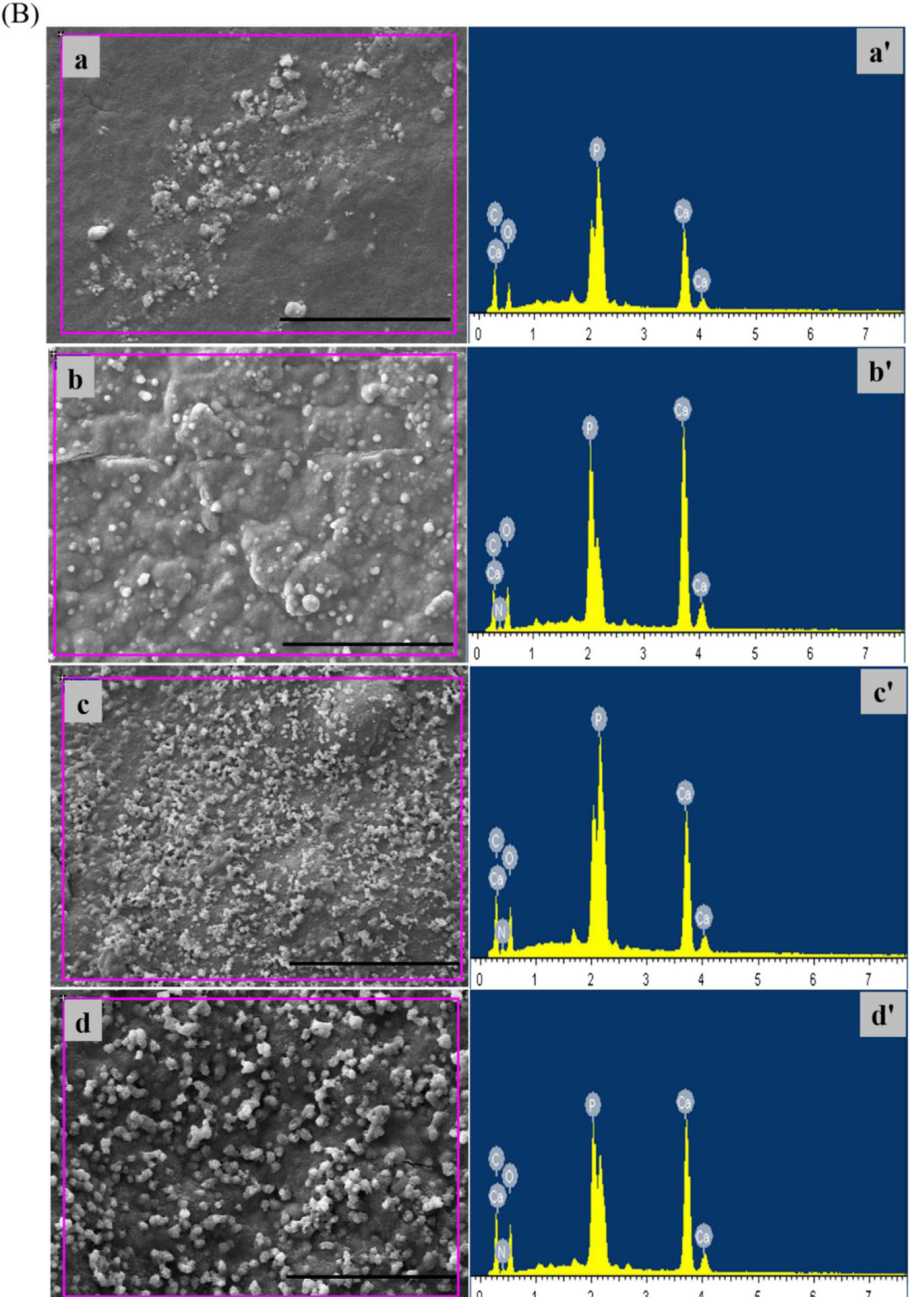
**A** SEM images of specimens (**a**–**e** referred PAA, 15%n-ACP-cit/PAA, 30%n-ACP-cit/PAA, 45%n-ACP-cit/PAA, and 55%n-ACP-cit/PAA) before and after (**a´–e**´ referred PAA, 15%n-ACP-cit/PAA, 30%n-ACP-cit/PAA, 45%n-ACP-cit/PAA, and 55%n-ACP-cit/PAA) soaking in SBF solution for 7 days; **B** EDX spectra of the composites surfaces (**a**, **a**’ referred 15%n-ACP-cit/PAA, **b**, **b**’ referred 30%n-ACP-cit/PAA, **c**, **c**’ referred 45%n-ACP-cit/PAA, and **d**, **d**’ referred 55%n-ACP-cit/PAA) after soaking in SBF solution for 7 days. Scale bar: 100 μm

SEM images of the surface morphology of PAA and n-ACP-cit/PAA composites before and after SBF soaking for a week are shown in Fig. 4A. Compared with initial surface (Fig. 4A(a–e)) and post-soaking (Fig. 4A(a´–e´)) of the n-ACP-cit/PAA composites, it revealed that a pronounced change had occurred, all the n-ACP-cit/PAA surfaces were covered by a layer of newly formed porous loose spherical apatite, and with the increase of n-ACP-cit in the composite, the amount of apatite became more and more. While there was no newly formed substance on PAA, which fully indicated that the addition of n-ACP-cit greatly improved the biological activity of the composite material in vitro. The corresponding composition analysis of the deposited particles in n-ACP-cit/PAA composites had also been done by EDX, as shown in Fig. 4B and Table 1. On the surface of the n-ACP-cit/PAA composite, Ca and P peaks appeared after SBF soaking for 7 days (Fig. 4B). In addition, it can be seen from Table 1, with the increase of content of n-ACP-cit in the composite material, the Ca/P molar ratio of apatite particles deposited on the surface of the composite material increased from 1.57 ± 0.03 to 1.70 ± 0.02 of 55 wt.% n-ACP-cit/PAA, among which, the Ca/P molar ratio of the apatite-like layer deposited by 45%n-ACP-cit/PAA composite material was closest to the theoretical Ca/P ratio of HA. The above results indicated that the addition of n-ACP-cit/PAA effectively improved the biological activity of PAA substrate in vitro. It could be inferred that the deposited particles were apatite particles.

**Table 1:** Atom percentages of different elements and the Ca/P molar ratios for the surfaces of specimens soaking in SBF solution for 7 days

Samples	Element type, atomic percentage, and Ca/P molar ratio					
	C	O	N	Ca	P	Ca/P mole ratio
PAA	50.59 ± 0.23	35.63 ± 0.19	13.78 ± 0.14	–	–	–
15%	44.86 ± 0.12	33.56 ± 0.11	10.04 ± 0.22	7.05 ± 0.21	4.49 ± 0.05	1.57 ± 0.03
30%	42.02 ± 0.23	36.05 ± 0.13	10.97 ± 0.18	6.76 ± 0.29	4.20 ± 0.07	1.61 ± 0.04
45%	39.33 ± 0.17	37.21 ± 0.16	12.11 ± 0.15	7.08 ± 0.27	4.27 ± 0.03	1.66 ± 0.05
55%	41.68 ± 0.19	34.97 ± 0.10	12.30 ± 0.14	6.96 ± 0.18	4.09 ± 0.06	1.70 ± 0.02

Values are expressed as mean ± SD (*n* = 3 for each sample).

### Cytocompatibility in vitro

#### Cell proliferation and morphology

**Fig. 5:**
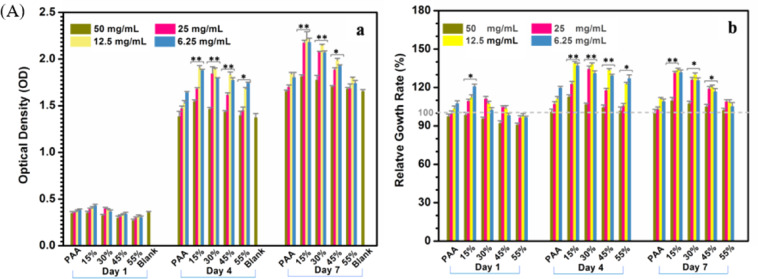

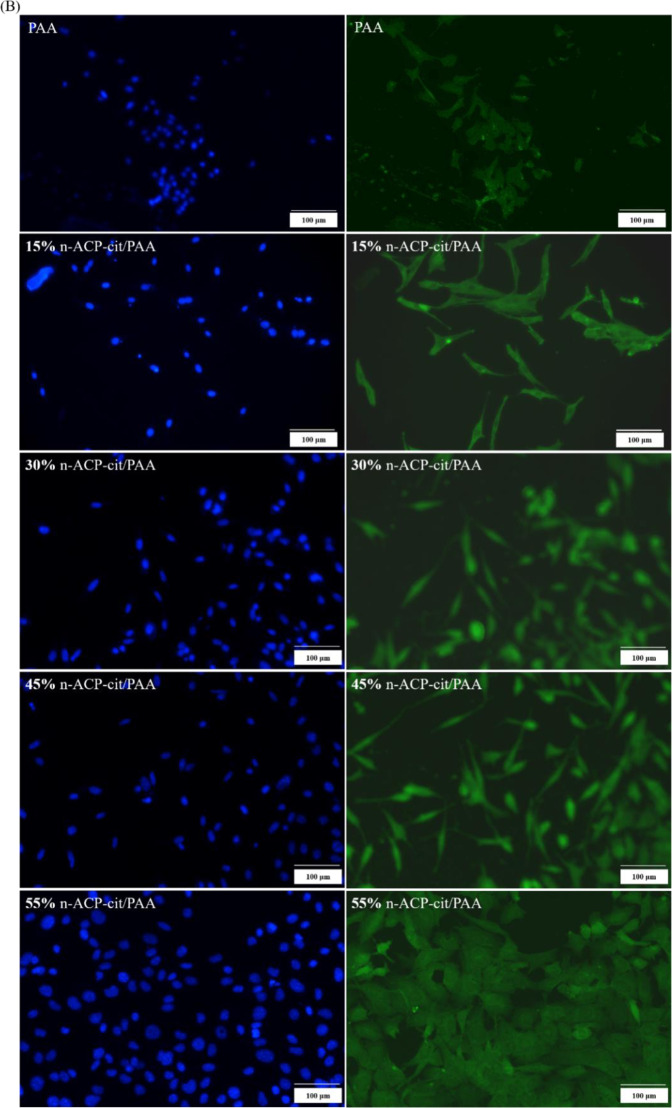
**A** The cell viability of mBMSCs cultured with PAA and n-ACP-cit/PAA composites at 1, 4, and 7 days (**a** the OD values; **b** the relative growth rate, *p < 0.05, **p < 0.01, compared with PAA). **B** The morphologies of mBMSCs cultured with PAA and n-ACP-cit/PAA composites for 3 days (the one on the left side were cell nucleus, the one on the right side were cytoplasm)

The cell proliferation on PAA and the n-ACP-cit/PAA composites at 1, 4, and 7 days using the CCK-8 assay was carried out. The obtained results were displayed in a bar graph of OD-culture time (Fig. 5A(a)) and the RGR (Fig. 5A(b)). In Fig. 5A, it was not hard to see that the cell proliferation rate of appropriate concentration of the leach liquor showed an increasing trend with the extension of culture time. When the leach liquor concentration of n-ACP-cit/PAA composite was 50 mg/ml and the cells were cultured for 1 day, the OD values between the composites and PAA were no significant difference. However, when the concentration leach liquor was the same, the content of n-ACP-cit in the composite increased gradually, the OD values showed a trend of increasing firstly and then decreasing slightly for culturing at 4 and 7 days. The morphology of mBMSCs co-cultured with the composites was flat, spindle-shaped and dilated, with clear frame and obvious antennae (Fig. 5B). With the increase of n-ACP-cit content in the composites, the cells were polygonal and cluster-like, and the number of them gradually increased. These results indicated that n-ACP-cit/PAA composites were not cytotoxic and could provide a good culture condition for the cells.

#### Osteogenesis of mBMSCs

**Fig. 6:**
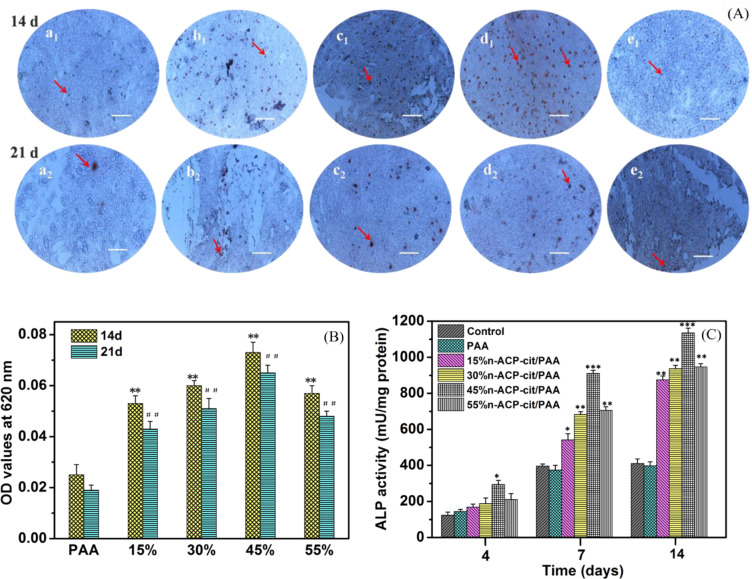
Qualitative (**A**) and quantitative (**B**) analysis of Alizarin Red Staining for PAA and n-ACP-cit/PAA composites: (**a**_**1**_, **a**_**2**_) PAA, (**b**_**1**_, **b**_**2**_)15%n-ACP-cit/PAA, (**c**_**1**_, **c**_**2**_) 30%n-ACP-cit/PAA, (**d**_**1**_, **d**_**2**_) 45%n-ACP-cit/PAA, and (**e**_**1**_, **e**_**2**_) 55%n-ACP-cit/PAA at 14 and 21 days, respectively (the red arrow refers the calcium nodules, scale bar: 100 μm,**p < 0.01, compared with PAA at 14 days, ^##^*p* < 0.01, compared with PAA at 21 days). **C** The ALP activities of mBMSCs cultured with PAA and n-ACP-cit/PAA composites at 4, 7, and 14 days, ***p < 0.001

We observed the mineralized calcium deposition and nodules in all groups after 14 and 21 days culturing in Fig. 6A (qualitative analysis) and Fig. 6B (quantitative analysis). Compared with the PAA group, a large amount of bone-like nodules were found in n-ACP-cit/PAA composite groups. Among them, the number of calcium nodules in all materials after 14 days was higher than that at 21 days, and the calcium nodules in 45%n-ACP-cit/PAA was the most. Moreover, the quantitative analysis results were consistent with the results of qualitative analysis about stained calcium nodules, which illustrating that the composites obtain the potential ability of promotion in bone mineralization.

The ALP activities of the mBMSCs cultured with PAA and n-ACP-cit/PAA composites shown in Fig. 6C were applied to evaluate the potential osteoblastic differentiation of mBMSCs on the composites, and the tissue culture plate was the control group. The ALP activity on the n-ACP-cit/PAA composites was significantly higher than that on PAA, and the 45%n-ACP-cit/PAA group showed the most potential ALP activity. Such an improvement could be associated with the incorporation of the n-ACP-cit powder phase into the PAA matrix, enhancing the differentiation ability of osteoblasts.

#### Real-time quantitative PCR

**Fig. 7:**
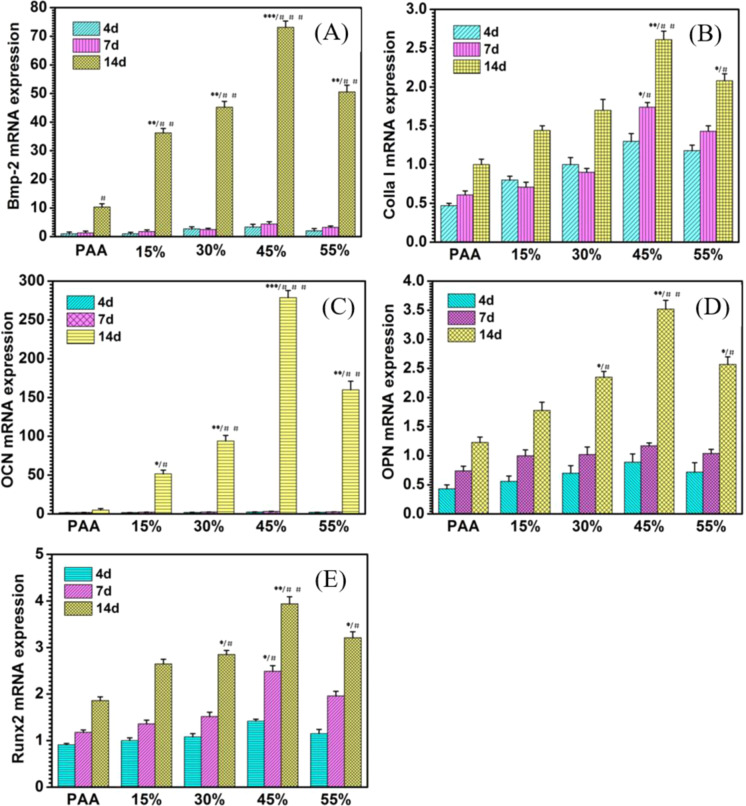
Representative genes expressed by PAA and n-ACP-cit/PAA composites cultured with mBMSCs for 4, 7, and 14 days (Bmp-2 (**A**), Colla I (**B**), OCN (**C**), OPN (**D**), and Runx-2 (**E**) (***p < 0.001, **p < 0.01, *p < 0.05, compared with PAA at 14 days; ^###^*p* < 0.001, ^##^*p*< 0.01, ^#^*p* < 0.05, compared with the corresponding composites at 4 and 7 days)

The gene expression levels of osteogenesis-related factors, Bmp-2, Colla I, OCN, OPN, and Runx-2 required for pre-osteoblast differentiation into mature osteoblasts were detected to investigate the osteogenic potential of the composites (Fig. 7). After induction for 4, 7, and 14 days, the mRNA expression levels of Bmp-2 (Fig. 7A), Colla I (Fig. 7B), OCN (Fig. 7C), OPN (Fig. 7D), and Runx-2 (Fig. 7E) in the composites were significantly higher than those in the PAA group, especially for culturing for 14 days, the obvious significant difference appeared. The result demonstrated that the addition of n-ACP-cit powder could enhance osteogenic activity of mBMSCs.

### Biocompatibility in vivo

**Fig. 8:**
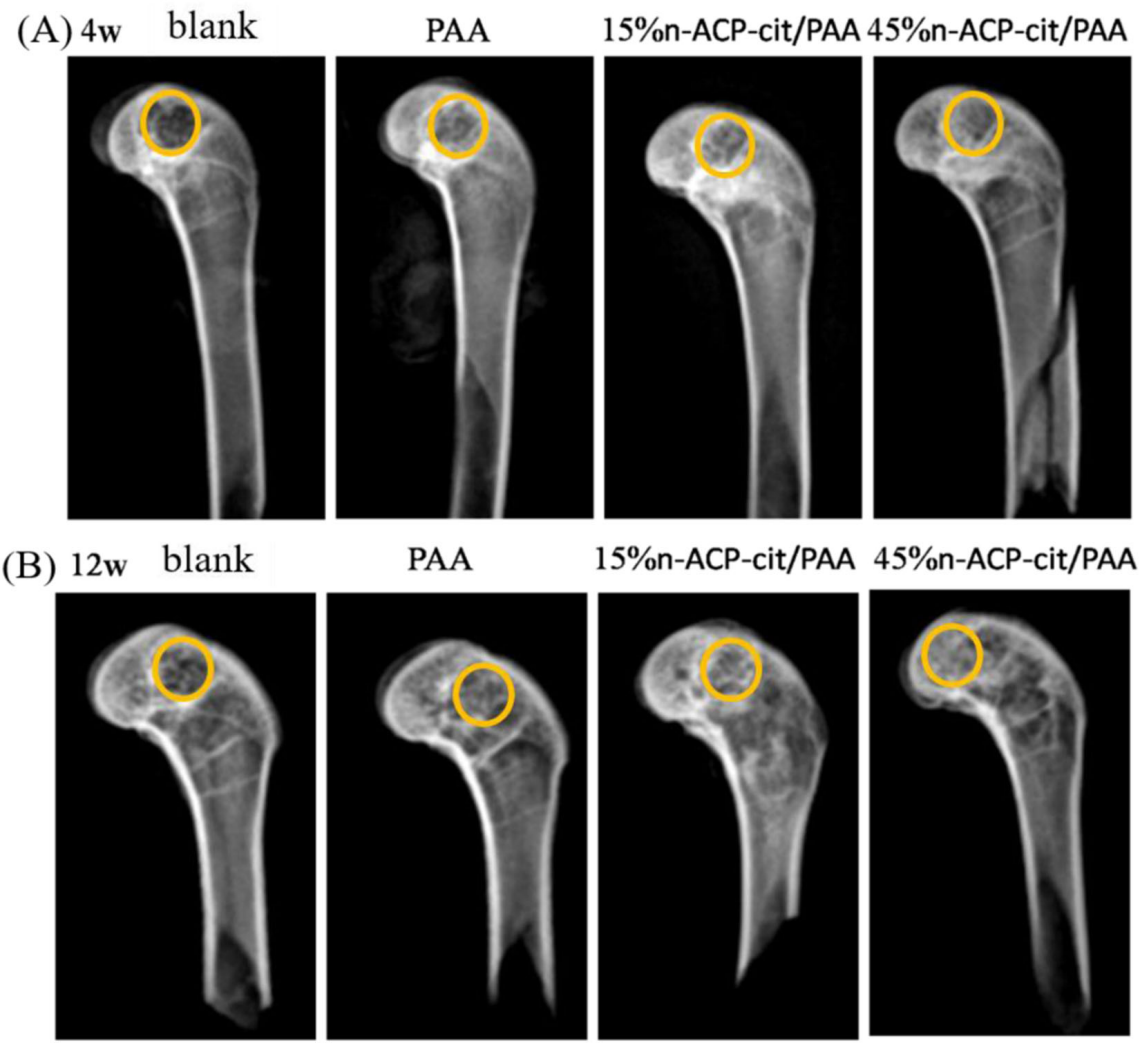
X-ray observation of all femoral specimens in rabbits at 4 weeks (**A**) and 12 weeks (**B**)

The X-ray images of the rabbit femur samples at 4 weeks (A) and 12 weeks (B) are shown in Fig. 8. At postoperative 4 and 12 weeks, the peripheral bone tissues surrounding the n-ACP-cit/PAA had no significant osteolysis, absorption, necrosis, or osteomyelitis. After surgery for 4 weeks, irregular area of bone defect could be seen at the external condyle of femur, and there was a small amount of low-density callus formed around the bone defect area. Over time, the bone mineral density of the implanted biomaterial of n-ACP-cit/PAA was consistent with the bone density of the peripheral tissues. Especially, the light transmission area at the external femoral condyle of 45%n-ACP-cit/PAA composite was significantly reduced, and the morphology of femoral condyle had been basically recovered.

**Fig. 9:**
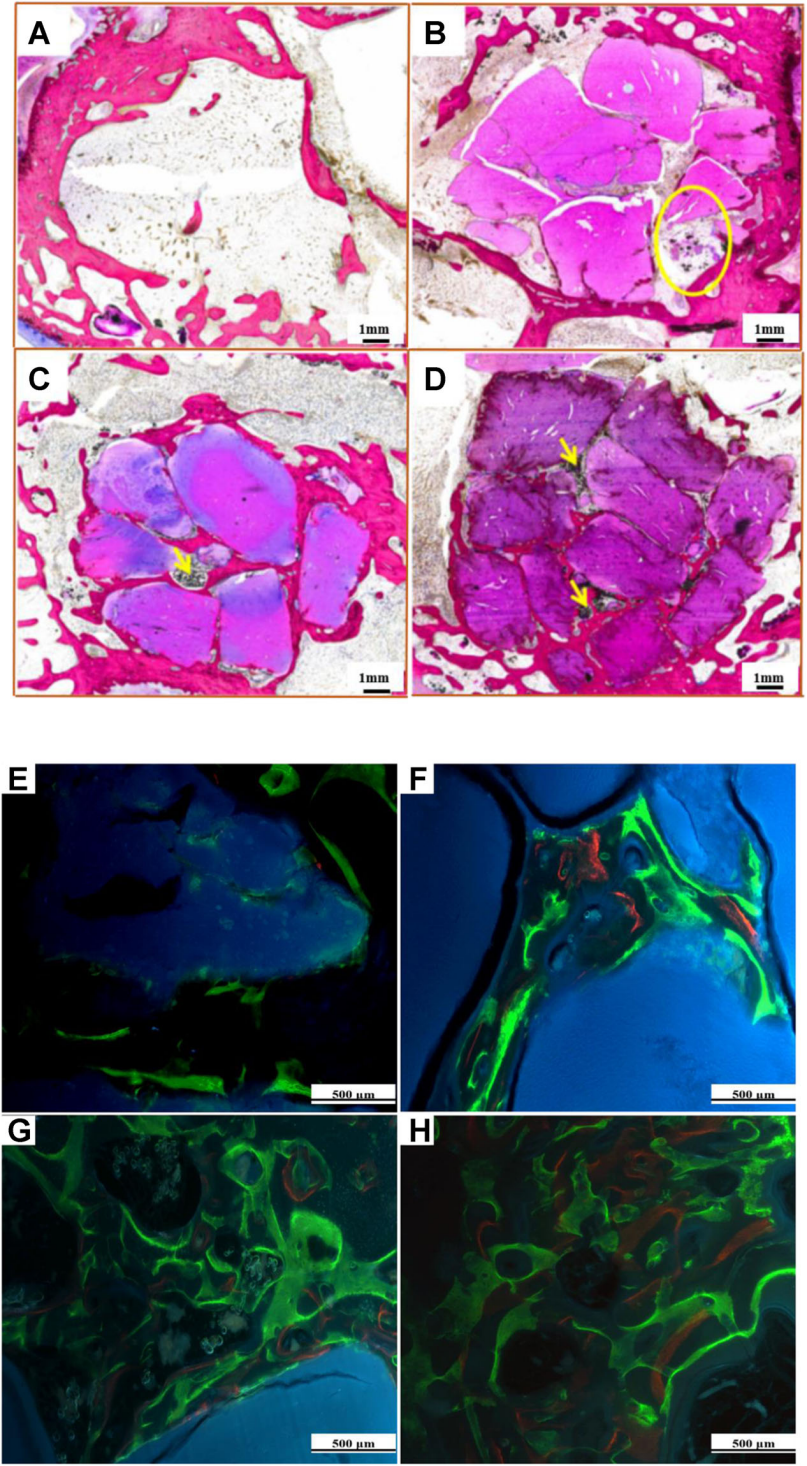
Histologic morphology (**A**–**D**) and fluorescence observations (**E**–**H**) of blank (**A**, **E**), PAA (**B**, **F**), 15%n-ACP-cit/PAA (**C**, **G**), and 45%n-ACP-cit/PAA (**D**, **H**) composites after 12-week femoral condyle implantation in rabbit

Figure 9a–d shows the histologic morphologies of all the samples after surgery at 12 weeks. Evidential bulk degradation or cell-mediated resorption of implants was not observed from histological observation. There was no inflammation found adjacent to the implants. In the composite’s groups, the new bone tissue began to grow into the material clearly, and the appearance of new bone trabeculae was observed (the yellow arrow). This indicated the addition of n-ACP-cit significantly in promoting the osteogenic activity of PAA material.

Calcein and dimethoxy phenol orange deposition were observed in bone formed in the experimental rabbits (Fig. 9e–h), indicating that bone formation started in all groups. The area of deposited color was significantly increased in composite groups, showing that more new bone formation in the implanted tissues, and the production of new bone by 15%n-ACP-cit/PAA and 45%n-ACP-cit/PAA were significantly increased between 6 and 9 weeks. Moreover, 45%n-ACP-cit/PAA had the most color deposition at all time points, showing excellent osteogenic activity.

Micro-CT scanning was performed on the samples after 4 and 12 weeks, and the osteogenesis-related indicators of BV/TV, Tb. N, Tb. Th, and Tb. Sp in the bone graft area of each group are listed in Table 2. As shown in Table 2, the n-ACP-cit/PAA composite was superior to PAA and the blank group in all the indicators, along with the increased content of n-ACP-cit, all the indicators showed an upward trend. These results once again indicated that the n-ACP-cit/PAA composite had good osteogenic activity.

**Table 2:** The micro-CT results of each group implanted after 4 and 12 weeks

Time	Groups	BV/TV	Tb. N (mm^−1^)	Tb. Th (mm)	Tb. Sp (mm)
4 weeks	Blank	0.002 ± 0.001	0.067 ± 0.002	0.044 ± 0.005	1.117 ± 0.005
	PAA	0.010 ± 0.003	0.093 ± 0.005	0.063 ± 0.003	1.030 ± 0.006
	15%n-ACP-cit/PAA	0.038 ± 0.004	0.127 ± 0.007	0.086 ± 0.001	0.910 ± 0.004
	45%n-ACP-cit/PAA	0.054 ± 0.005	0.295 ± 0.003^b^	0.098 ± 0.002	0.635 ± 0.003^b^
12 weeks	Blank	0.018 ± 0.003	0.309 ± 0.002	0.115 ± 0.006	3.171 ± 0.002
	PAA	0.052 ± 0.002	0.367 ± 0.001	0.137 ± 0.003	2.906 ± 0.004^b^
	15%n-ACP-cit/PAA	0.066 ± 0.005^a,b^	0.749 ± 0.006^a,b^	0.146 ± 0.002^a^	1.403 ± 0.005
	45%n-ACP-cit/PAA	0.083 ± 0.004^a,b^	0.926 ± 0.003^a,b^	0.257 ± 0.004^a,b^	1.146 ± 0.003^b^

^a^*p* < 0.05, each sample compared at 4 and 12 weeks.

^b^*p* < 0.05, compared with blank or PAA group at the same time point.

## Discussion

The study investigated a novel n-ACP-cit/PAA composite combining the good degradability, superior bioactivity, and osteogenic activity of n-ACP-cit powder with the excellent properties of PAA matrix that is similar to the polypeptide architecture of natural collagen proteins [24]. Among the factors influencing the physicochemical and biological properties of the composites, the interface combination between the different components of the composites was significantly important [25]. The basic characterizations (XRD, FT-IR, and XPS) of the n-ACP-cit/PAA composites (Fig. 1) indicated the existence of citrate, phosphate, and PAA within the as-prepared composites. In detail, the decreased crystallinity of PAA in the composites due to the introduced n-ACP-cit powder may reduce the regularity of PAA molecular chain (Fig. 1A). In the FT-IR spectra (Fig. 1B), there were slight shifts of characteristic absorption peaks in the composites, which confirmed that there was a certain interface interaction between n-ACP-cit and PAA [10]. Compared with n-ACP-cit and PAA matrix, the binding energy of each component in the composite material had changed in different degrees (Supplementary Table 3), indicating a possible interaction between the PAA material and n-ACP-cit powder, such as the combination of -C=O, -NH-, and -COO^–^ groups in PAA with Ca^2+^ that presents in n-ACP-cit powder.

The other key factor that determined the clinical application of the biomaterials is biomechanical behaviors [26]. In this research, the mechanical properties could be adjusted by changing the n-ACP-cit content in the composites, shown in Fig. 2. The highest compressive, bending, and tensile strengths were 5.65 ± 0.22, 13.97 ± 0.79, and 12.85 ± 0.59 MPa, respectively, with 45 wt.% n-ACP-cit in the composite. Increased mechanical properties of the composites might be due to the filler reinforcing effect of n-ACP-cit and strong interfacial interaction facilitated the effective transmission of loads. This was consistent with some studies that improved the mechanical properties of polymers by adding inorganic materials [27]. But with 55 wt.% n-ACP-cit in the composite, the mechanical properties decreased, it might be due to the agglomeration of n-ACP-cit nanoparticle, which hinders the ductile flow of PAA chain.

The influence of degradation and pH values of the degradation solution was also significant one. The weight losses of the n-ACP-cit/PAA with different contents of n-ACP-cit powder in the SBF solution after 16 weeks soaking were 23.99, 26.11, 33.77, 36.01, and 23.79 wt.%, respectively. The hydrolysis of PAA and dissolution of n-ACP-cit powder, the dissolution of degraded small molecules, and oligomers are the main parts of the degradation process. For PAA, its hydrolysis is mainly due to the hydrolysis of amide bond (-CO-NH-), and the hydrolysis could be accelerated in acidic environment [28, 29]. Moreover, the formed apatite on the composites surface might be the main reason for the reduced degradation, especially for 55%n-ACP-cit/PAA composite. At the first week, the slight acidic degradation product of PAA resulted in relative lower pH value (6.96), while with the increasing content of n-ACP-cit in composite, n-ACP-cit began to dissolve to release Ca^2+^ and PO_4_^3–^ leading to the higher concentration of Ca and P ions in the solution and the higher pH value, which was helpful to the apatite deposition process. In reverse, the more deposited apatite, the higher the pH value. Finally, the pH values of composite materials were stable at the optimal pH range required by human physiological environment.

The bioactivity is an important index to measure artificial biomaterials before the clinical implantation [26]. The ability to form apatite reflects the interaction between bone tissue and biomaterial interface, which is beneficial to improve the cytocompatibility [30]. In this study, the SEM images of materials revealed that the surfaces of the composites were covered with some Ca-P layer with porous loose spherical structure after immersing in SBF solution for 7 days. During the process of apatite deposition, the Ca ion and citrate of n-ACP-cit would play an important role in the formation of apatite on the composites’ surface. Moreover, the citrate could adsorb or bind Ca^2+^ and form Ca^2+^ aggregates at the surface of n-ACP. Also, the Ca^2+^ will attract PO_4_^3–^ and deposited on the composites’ surface, among this dynamic process apatite nuclei was formed. Then the Ca and P ions in SBF solution were consumed to continuing forming apatite layer. In a whole, the formation of apatite layer belongs to a dissolution-binding-precipitation process [31]. In addition, the EDX data were also an indication of the formation of a new Ca-P layer. Based on the combination of these data, it is easy to find that the mineralization ability of the composites was increased with the addition of n-ACP-cit powder.

Biocompatibility is a critical element in the evaluation of a biomaterial [32, 33]. The cell viabilities result (Fig. 5A) illustrated that the introduction of n-ACP-cit could stimulate cell growth at relative lower concentrations. The morphology changes (Fig. 5B) of the co-cultured mBMSCs proved that n-ACP-cit/PAA composite has good cellular compatibility.

As we all know, bone formation is a molecular event that adjust by cellular in fine orchestrated way. Osteoblasts are derived from the step-by-step differentiation of bone marrow mesenchymal stem cells, whose main function is to generate bone tissue fibers and organic matrix [34]. Therefore, biomaterials for bone tissue repair are particularly important for promoting new bone formation, osteoblast differentiation, and osteogenic activity. To elucidate the effects of n-ACP-cit/PAA composites on the expression of proteins and genes associated with osteoblast differentiation of mBMSCs, we investigated the ALP activity assay (quantitatively), calcium deposition (qualitative and quantitative analysis), and qRT-PCR test. Compared with PAA group, the ALP activity and the expressions of osteogenic differentiation-related genes (Bmp-2, Colla I, OCN, OPN, and Runx-2) of the cells on n-ACP-cit/PAA composites were significantly higher. This illustrates that the composites could promote differentiation of mBMSCs owing to the introduction of n-ACP-cit with high bioactivity and osteogenic activity [35]. Moreover, the formed apatite on the composites’ surface was also favorable to promote the differentiation of osteoblasts [36]. In addition, the formation of calcium nodules and the improved expression of osteogenesis-related genes indicate that the incorporation of n-ACP-cit powder could control the mineralization of osteoid. In summary, these results demonstrated that the n-ACP-cit/PAA composites could be considered as a potential bone repair material as to the facilitated proliferation, differentiation of mBMSCs.

Last but not the least, the biocompatibility in vivo of n-ACP-cit/PAA composite material was further evaluated through the rabbit femoral condyle defect model. The X-ray images (Fig. 8), histologic morphology (Fig. 9A–D), and fluorescence analysis (Fig. 9E–H) of the femur samples at different time exhibited the sign of bone repair, especially after introduction of the n-ACP-cit into the composite. Moreover, the micro-CT results (Table 2) showed that the osteogenesis-related indicators of each group (BV/TV, Tb. N, Tb. Th, and Tb. Sp) in the bone graft area were higher than that of pure PAA. The as-mentioned results indicated that the addition of n-ACP-cit effectively improved the osteogenic ability and bone conduction activity of the composite.

## Conclusions

In this work, the modified n-ACP-cit/PAA composites were successfully fabricated by melting extrusion method. The incorporation of n-ACP-cit powder increased the degradation property. Mineralization ability of the as-prepared composites was greatly enhanced due to n-ACP-cit with ease degradability and high bioactivity. The results revealed that higher cell proliferation and better cell morphology appeared on the modified n-ACP-cit/PAA composites. In addition, the introduction of n-ACP-cit powder enhanced the osteogenic differentiation and activity of the composites compared with PAA in vitro and in vivo. Above all, it could be believed that the modified n-ACP-cit/PAA composites would be promising candidates as a novel osteoconductive composite material for bone repair.

## Supplementary information

Supplemental Materials
