# Supplementary material for: Biological evaluation of the modified nano-amorphous phosphate calcium doped with citrate/poly-amino acid composite as a potential candidate for bone repair and reconstruction
Source: J Mater Sci Mater Med. 2021 Jan 25;32(1):16. doi: 10.1007/s10856-020-06482-7 (PMC7829244; doi:10.1007/s10856-020-06482-7)
Supplement: Supplementary file 1 — Supplemental Materials [file 10856_2020_6482_MOESM1_ESM.docx]

Supplemental Materials

**Biological Evaluation of the modified Nano-Amorphous Phosphate Calcium Doped with Citrate/Poly-Amino Acid Composite as a Potential Candidate**

**for Bone Repair and Reconstruction**

Xiaomei Wang^1,2^, Dechuan Zhao^2^, Haohao Ren^1*^, Yonggang Yan^1*^, Shuyang Li^1^

1. College of Physics, Sichuan University, Chengdu 610064, China

2. Collaborative Innovation Center of Tissue Repair Material of Sichuan Province, College of Life Sciences, China West Normal University, Nanchong 637009, China

* Correspondences: [yan_yonggang@vip.163.com](mailto:yan_yonggang@vip.163.com); renhaohao@scu.edu.cn

**Table 1** Primers sequence used for RT-Qpcr

| Name of the primers | Sequence of the primers | Length of the fragment (bp) | Annealing temperature  (℃) |
| --- | --- | --- | --- |
| M-β-actin-S | GTGACGTTGACATCCGTAAAGA | 287 | 60 |
| M-β-actin-A | GTAACAGTCCGCCTAGAAGCAC |  | 60 |
| M-spp1(rz)-S | TTTCACTCCAATCGTCCCTACA | 237 | 60 |
| M-spp1(rz)-A | CTGCCCTTTCCGTTGTTGTC |  | 60 |
| M-Bmp2-S | CGAATTTGAGTTGAGGCTGCTC | 234 | 60 |
| M-Bmp2-A | GCCGTTTTCCCACTCATCTCT |  | 60 |
| M-col1a1-S | AAGAAGCACGTCTGGTTTGGAG | 175 | 60 |
| M-col1a1-A | GGTCCATGTAGGCTACGCTGTT |  | 60 |
| M-runx2-S | AGCGGACGAGGCAAGAGTTT | 219 | 60 |
| M-runx2-A | AGGCGGGACACCTACTCTCATA |  | 60 |
| M-bglap-S | GGAGGGCAATAAGGTAGTGAACAG | 119 | 60 |
| M-bglap-A | ATAGCTCGTCACAAGCAGGGT |  | 60 |

**Table 2** The injection parameters of fluorochrome labeling

| The specific parameters | calcein | xylenol orange | calcein |
| --- | --- | --- | --- |
| manufacturer | Sigma aldrich, R&D  Japan | Aladdin, Indicator  China | Sigma aldrich, R&D  Japan |
| solvent | 2% NaHCO_3_ | 1% NaHCO_3_ | 2% NaHCO_3_ |
| pH | 6-8 | 6-8 | 6-8 |
| concentration | 5 mg/mL | 50 mg/mL | 5 mg/mL |
| dose | 10 mg/kg (=2 mL/kg) | 100 mg/kg (=2 mL/kg) | 10 mg/kg (=2 mL/kg) |
| Injection points | 1 weeks | 2 weeks | 3 weeks |
| Injection points | 6 weeks | 9 weeks | 6weeks |

**Table 3** Binding energies C1s, O1s, N1s and Ca2p and P2p of n-ACP-cit, PAA and

55%n-ACP-cit/PAA composite

| Samples |  | Binding energy of the elements（eV） | | | | | | |
| --- | --- | --- | --- | --- | --- | --- | --- | --- |
|  | C1s | | C1s | O1s | N1s | Ca2p3/2 | Ca2p1/2 | P2p |
| n-ACP-cit | 283.98 | | 287.13 | 531.22 | / | 346.74 | 350.15 | 132.58 |
| PAA | 286.99 | | 290.01 | 533.01 | 401.84 | / | / | / |
| 55%n-ACP-cit/PAA | 286.30 | | 289.33 | 533.34 | 401.30 | 348.69 | 351.98 | 134.90 |


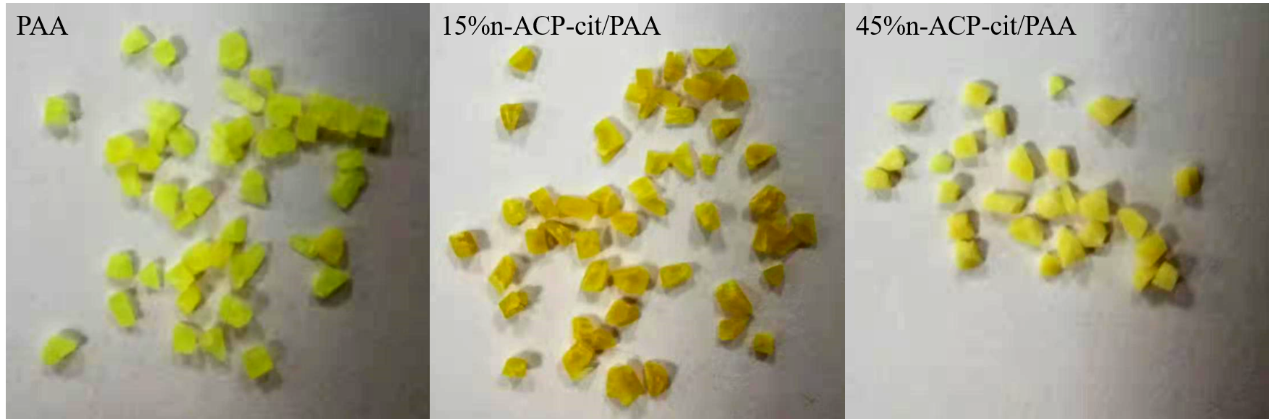


**Figure 1** Photos of the implanted materials


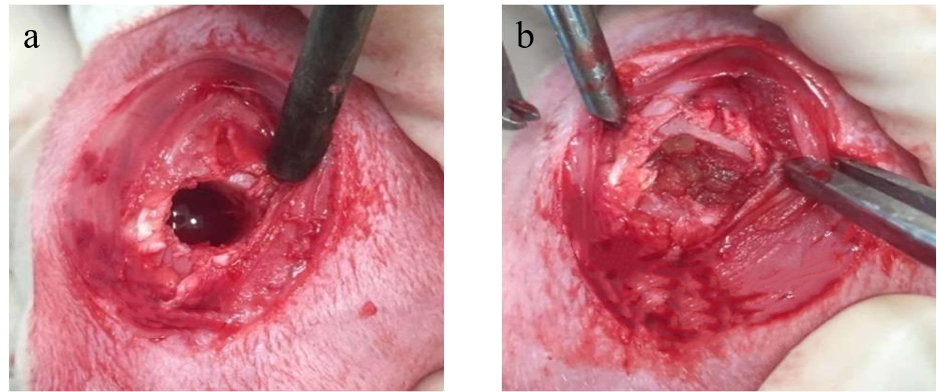


**Figure 2** Photos of the operation process in femoral condyle before (a) and after (b)
